# Elite controllers long-term non progressors present improved survival and slower disease progression

**DOI:** 10.1038/s41598-022-19970-3

**Published:** 2022-09-29

**Authors:** Laura Capa, Rubén Ayala-Suárez, Humberto Erick De La Torre Tarazona, Juan González-García, Jorge del Romero, José Alcamí, Francisco Díez-Fuertes

**Affiliations:** 1 AIDS Immunopathogenesis Unit, National Center of Microbiology, Instituto de Salud Carlos III, Madrid, Spain; 2 Centro de Investigación Biomédica en Red de Enfermedades Infecciosas (CIBERINFEC), Madrid, Spain; 3 Departamento de Biomedicina y Biotecnología, Universidad de Alcalá, Alcalá de Henares, Spain; 4 Unidad de VIH, Servicio de Medicina Interna, Hospital Universitario La Paz, Idipaz, Madrid, Spain; 5 Centro Sanitario Sandoval, Hospital Clínico San Carlos, IdISSC, Madrid, Spain; 6 Infectious Diseases Unit, IBIDAPS, Hospital Clinic, University of Barcelona, Barcelona, Spain

**Keywords:** Hepatitis, HIV infections, HIV infections

## Abstract

Different phenotypes exhibiting no evidences of disease progression have been described in ART-naïve HIV-1 positive individuals. Long-term non progressors (LTNP) and elite controllers (EC) are low frequent examples of immunological and virological control in HIV-1 positive subjects, respectively. The combination of both phenotypes is even less frequent and studied despite being considered as models of HIV-1 functional cure. A multicenter, prospective study in retrospect including clinical and epidemiological data collected from 313 LTNP of 21 Spanish hospitals was carried out. LTNPs maintaining CD4+ T cell counts over 500 cells/µl and viral loads (VL) under 10,000 copies/mL for at least 10 years in the absence of antiretroviral therapy were followed for a median of 20.8 years (IQR = 15.6–25.5). A 52.1% were considered EC (undetectable VL) and LTNP (EC-LTNP) and a total of 171 (54.8%) and 42 (13.5%) out of the 313 participants maintained LTNP status for at least 20 and 30 years, respectively. EC-LTNP showed lower CD4+ T cell count loss (9.9 vs 24.2 cells/µl/year), higher CD4/CD8 ratio (0.01 vs − 0.09 in ratio), and lesser VL increase (no increase vs 197.2 copies/mL/year) compared with LTNPs with detectable VL (vLTNP). Survival probabilities for all-cause mortality at 30 years from HIV + diagnosis were 0.90 for EC-LTNP and 0.70 for vLTNP (*p* = 2.0 × 10^−3^), and EC-LTNP phenotype was the only factor associated with better survival in multivariate analyses (HR = 0.28; 95% CI 0.10–0.79). The probability to preserve LTNP status at 30 years was 0.51 for EC-LTNP and 0.18 for vLTNP (*p* < 2.2 × 10^−16^). Risk factors associated to the loss of LTNP status was: higher age at diagnosis and the increase of VL, whereas the increase of CD4+ T cell counts and CD4/CD8 ratio, the initial EC-LTNP phenotype and HCV coinfection were protective factors. EC-LTNP phenotype was associated with improved survival and slower disease progression compared with other phenotypes of LTNP. EC-LTNP individuals represent one of the most favorable phenotypes of immune activation against HIV-1 found in nature and, therefore, are strong candidates to be considered a model of functional cure of HIV-1 infection.

## Introduction

The Spanish HIV/AIDS Research Network (RIS) currently brings together 37 Spanish research groups from 11 of Spain’s 17 regions, and includes basic, clinical and epidemiology research. The network has generated unique structures in our country, among them, the cohorts and the biobank are the most noteworthy legacy of the network^1^. The cohorts of people living with HIV (PLWH) with extreme phenotypes provide essential clues to understand HIV immunopathogenesis, they can control viral replication longer or have a delayed progression to immune deficiency or, on the contrary, can develop atypical rapid progression to AIDS^2,3^. One of the RIS consolidated cohorts of extreme phenotypes of special relevance due to the immunologic and virologic characteristics of the participants, is the Long-Term Non-Progressors Cohort (LTNP-RIS). LTNP-RIS participants are defined as those maintaining high levels of CD4 cells (above 500 cells/µl) and low levels of VL (below 10,000 copies/mL) for more than 10 years without antiretroviral treatment. This cohort can help us to find correlates of HIV-disease protection, which will be important for the development of future HIV vaccines and immunotherapies. In addition, as new treatment guidelines recommend earlier treatment initiation, this cohort is especially valuable due to the difficulty to identify new individuals with this phenotype.

The LTNP-RIS Cohort was created in 2006, and, at present, gathers clinical information and biological samples^1^ of participants from 21 hospitals located in 15 different cities from 9 Spanish regions. These participants are classified in three groups: elite LTNP (EC-LTNP) with undetectable VL in addition to the LTNP characteristics, viremic LTNP (vLTNP) with VL between 50 and 10.000 copies/mL in addition to the LTNP characteristics, and ExLTNP who have lost LTNP criteria.

This article describes the demographic and clinical characteristics of the subjects enrolled in the LTNP-RIS Cohort, and the differential characteristics of EC-LTNP and vLTNP participants.

## Patients and methods

### Patients

LTNP participants are defined as those maintaining CD4 cells above 500 cells/µl (in at least 75% of all determinations) and VL below 10,000 copies/mL for more than 10 years since HIV + diagnosis without antiretroviral treatment. When participants are included in the cohort, they are classified as EC-LTNP or vLTNP. EC-LTNP have undetectable VL in at least 75% of all samples in addition to the above LTNP characteristics. On his part, vLTNP have VL between 50 and 10.000 copies/mL in > 25% of the laboratory data in addition to the above LTNP characteristics.

Participants can change their LTNP status along time as the result of the progression of HIV-1 infection. An EC-LTNP is reclassified to viremic LTNP when VL is between 50 and 10.000 copies/mL during at least one year, regardless the number of laboratory tests. In addition, vLTNP or EC-LTNP can become an ExLTNP if, during at least one year, CD4 cells are below 500 cells/µl, or VL is above 10.000 copies/mL, or both (regardless the number of laboratory tests). Starting treatment is another reason to define an ExLTNP. The cohort keeps gathering clinical information of those participants who have lost LTNP criteria.

### Statistical analyses

We used the participants’ reported date of first positive HIV test as date of diagnosis. For those individuals who did not report this date, we used the date included in the medical record. A Kaplan–Meier method was used to estimate the probability of survival and progression-free survival for the individuals included in the cohort, censoring the follow-up at the date when individuals were last assessed for any laboratory measurement (CD4 T cell count or HIV RNA load) or the initiation of antiretroviral therapy, whichever occurred first. Time to event was defined as time from HIV diagnosis to exitus, for survival analysis, and time from HIV diagnosis to the loss of LTNP status, for the progression-free survival analysis. We compared the differences in survival curves obtained by age at HIV + diagnosis, sex, transmission route, progression phenotype at the inclusion in the cohort, CD4 T cell count loss, VL trend over time, CD4/CD8 ratio loss, hepatitis C virus (HCV) coinfection and evidence of progression (i.e. changes in EC-LTNP or vLTNP status) using a log-rank test. Observed to expected numbers of events at each time point were compared over the follow-up period. We calculate the relative hazard of exitus and loss of LTNP phenotype using univariate Cox proportional hazards regression model using the following covariates: age at HIV + diagnosis, transmission route, progression phenotype at the inclusion in the cohort, CD4 T cell count loss, VL trend over time, CD4/CD8 T cell count loss and HCV coinfection. In these cases, only statistically significant covariates in the univariate analysis were kept for the multivariate Cox proportional hazards regression model to estimate both the relative hazards of death and the loss of LTNP phenotype. In the latter, evidence of progression was not included as a covariate in the model because it defines progression itself.

### Ethics approval and consent to participate

Patients gave their informed consent for the use of clinical data in the Spanish LTNP cohort including blood donation in the HIV Biobank. These agreements were approved by the Ethical Committee of Hospital Gregorio Marañón in Madrid. All research was performed in accordance with the relevant guidelines and regulations.

## Results

### Overview of LTNP-RIS cohort

LTNP-RIS is currently composed of 313 HIV + individuals, including 163 EC-LTNP (52.1%) and 150 vLTNP (47.9%). Almost all participants of LTNP-RIS cohort were from Spanish origin (95.5%), two thirds were men (64.9%), and the most frequent route of infection was injection-drug use (75.4%), followed by heterosexual transmission (14.6%) and sex between men (6.8%). The median age at HIV + diagnosis was 25.6 years (IQR = 22.5–30.5). Among those who reported academic level, half reported primary education (52.4%), followed by secondary education (27.3%), tertiary education (11.2%) and without any level (9.1%). Nearly half of participants required antiretroviral therapy at some time (44.4%), including a 42.9% of men and 47.3% of women (ART was initiated during pregnancy in 15 women). A total of 62.0% of vLTNP have required ART against a 28.2% of EC-LTNP. The main characteristics of the participants are summarized in Table 1.

**Table 1:** Main characteristics of LTNP-RIS cohort.

	Total (n = 313)	EC-LTNP (n = 163)	vLTNP (n = 150)
**Median follow-up without therapy in years** (IQR)	20.3 (15.2–25.2)	22.8 (17.3–28.0)	18.2 (14.6–22.4)
**Median age at HIV + diagnosis** (IQR)	25.6 (22.5–30.5)	25.3 (22.0–30.6)	26.3 (23.0–30.5)
**Sex**			
Male	203 (64.9%)	97 (59.5%)	106 (70.7%)
Female	110 (35.1%)	66 (40.5%)	44 (29.3%)
**Nationality**			
Spaniards	299 (95.5%)	155 (95.1%)	144 (96.0%)
Other	14 (0.5%)	8 (4.9%)	6 (4.0%)
**Academic education**			
None	17 (9.1%)	10 (10.1%)	7 (7.9%)
Primary education	98 (52.4%)	49 (49.5%)	49 (55.7%)
Secondary education	51 (27.3%)	27 (27.3%)	24 (27.3%)
Terciary education	21 (11.2%)	13 (13.1%)	8 (9.1%)
**HCV coinfection**			
Yes	259 (82.7%)	140 (85.9%)	119 (79.3%)
**Transmission route**			
Sex between men	21 (6.8%)	5 (3.1%)	16 (10.7%)
Heterosexual	45 (14.6%)	25 (15.6%)	20 (13.3%)
Injection drug use	236 (75.4%)	125 (78.1%)	111 (74.5%)
Other	7 (2.3%)	5 (3.1%)	2 (1.3%)
**Antiretroviral therapy**	139 (44.4%)	46 (28.2%)	93 (62.0%)
**Loss of non-progression during follow-up**			
EC-LTNP during follow-up	–	97 (59.5%)	–
EC-LTNP to vLTNP	–	13 (8.0%)	–
EC-LTNP to vLTNP to ExLTNP	–	22 (13.5%)	–
EC-LTNP to ExLTNP	–	31 (19.0%)	–
vLTNP during follow-up	–	–	52 (34.7%)
vLTNP to ExLTNP	–	–	98 (65.3%)
**CD4+ T cell count characteristics**			
Total median number of measurements since HIV + diagnosis	19 (11–30)	19 (10–31)	20 (12–30)
Median time in days between measurements	182.0 (132.0–245.0)	186.0 (137.1–254.4)	178.0 (129.5–237.0)
**HIV RNA load characteristics**			
Total median number of measurements since HIV + diagnosis	18 (10–28)	17.5 (10.0–29.0)	18.0 (11.0–27.0)
Median time in days between measurements	180.5 (132.0–249.0)	187.0 (139.0–251.5)	175.0(125.5–234.9)
**CD4/CD8 cell count characteristics**			
Total median number of measurements since HIV + diagnosis	11 (0–20)	11 (1–19.8)	10 (0–20)
Median time in days between measurements	185.5 (141.8–246.8)	192.0 (152.5–263.3)	181.0 (136.2–234.2)

### Long-term HIV-1 control without signs of disease progression

The LTNP status was preserved in the LTNP-RIS cohort for a median time of 20.8 years (IQR = 15.6–25.5), with median times of 23.6 (IQR = 18.7–28.8) and 18.2 (IQR = 14.9–22.2) for EC-LTNP and vLTNP, respectively. More than half of participants (n = 171) have preserved LTNP status for at least 20 years (54.8%). This proportion fall to 13.5% for LTNP maintaining the phenotype for at least 30 years (n = 42). In the case of these 42 LTNP retaining LTNP status for more than 30 years, three quarters was initially classified as EC-LTNP (n = 31; 73.8%) and the rest was initially classified as vLTNP (n = 11; 26.2%). These 42 participants maintain the LTNP status a median time of 32.5 years (IQR = 31.3–33.3) and two of them have exceeded the barrier of 35 years.

### HCV coinfection

**Figure 1:**
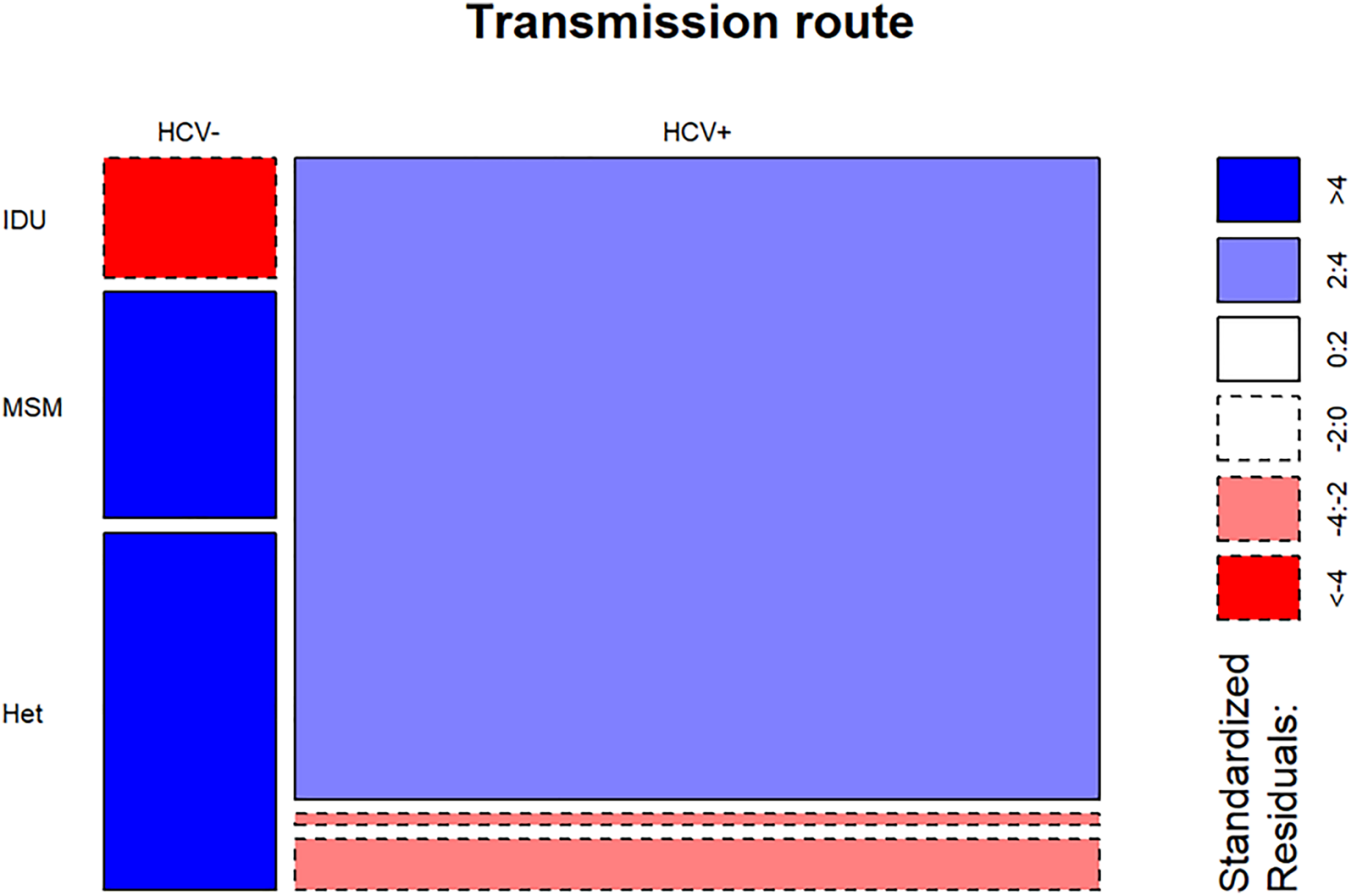
HCV coinfection by transmission route. The size of each rectangle is proportionate to the size of total number of individuals in that combination of HCV coinfection status and HIV transmission route. The color of each rectangle indicates how much that combination of variables differs from what would be expected if the two variables were unrelated. Blue indicates higher proportions than expected and red indicates lower proportions than expected.

A high proportion of individuals included in the cohort were diagnosed as HCV positive during follow-up (82.7%), only 14.7% were not coinfected, and 8 participants (2.6%) did not have serological data. The proportion of coinfected men was 85.7% against the 77.3% observed in women. Comparing the initial phenotypes of progression, a 85.9% of EC-LTNP and a 79.3% of vLTNP were coinfected. Strong differences were found comparing the route of transmission, since almost all IDU were coinfected (96.2%) in comparison to sexual transmission, where these frequencies dropped to 40.0% for heterosexual transmission and to 19.1% in MSM (*p* < 2.2 × 10^−16^) (Fig. 1). No differences were found comparing the proportions of HCV + (44.4%) and HCV- (45.7%) requiring ART.

### CD4 cell counts and CD4/CD8 during follow-up

The median CD4 counts for EC-LTNPs was 817.7 cells/μl (IQR = 691.7–913.0 cells/μl), whereas for vLTNP was 700.0 cells/μl (IQR = 598.5–832.5 cells/μl; *p* = 1.17 × 10^−4^). The median variation of CD4 counts in participants primarily classified as EC-LTNP and vLTNP was − 9.9 (IQR from − 21.1 to 9.0) and − 24.2 (IQR from − 42.3 to − 11.7) cells/μl/year, respectively (*p* = 1.2 × 10^−7^). Among EC-LTNP who maintain their status during the follow-up, the median variation of CD4 counts was 0.49 cells/μl/year (IQR from − 14.5 to 16.5).

**Figure 2:**
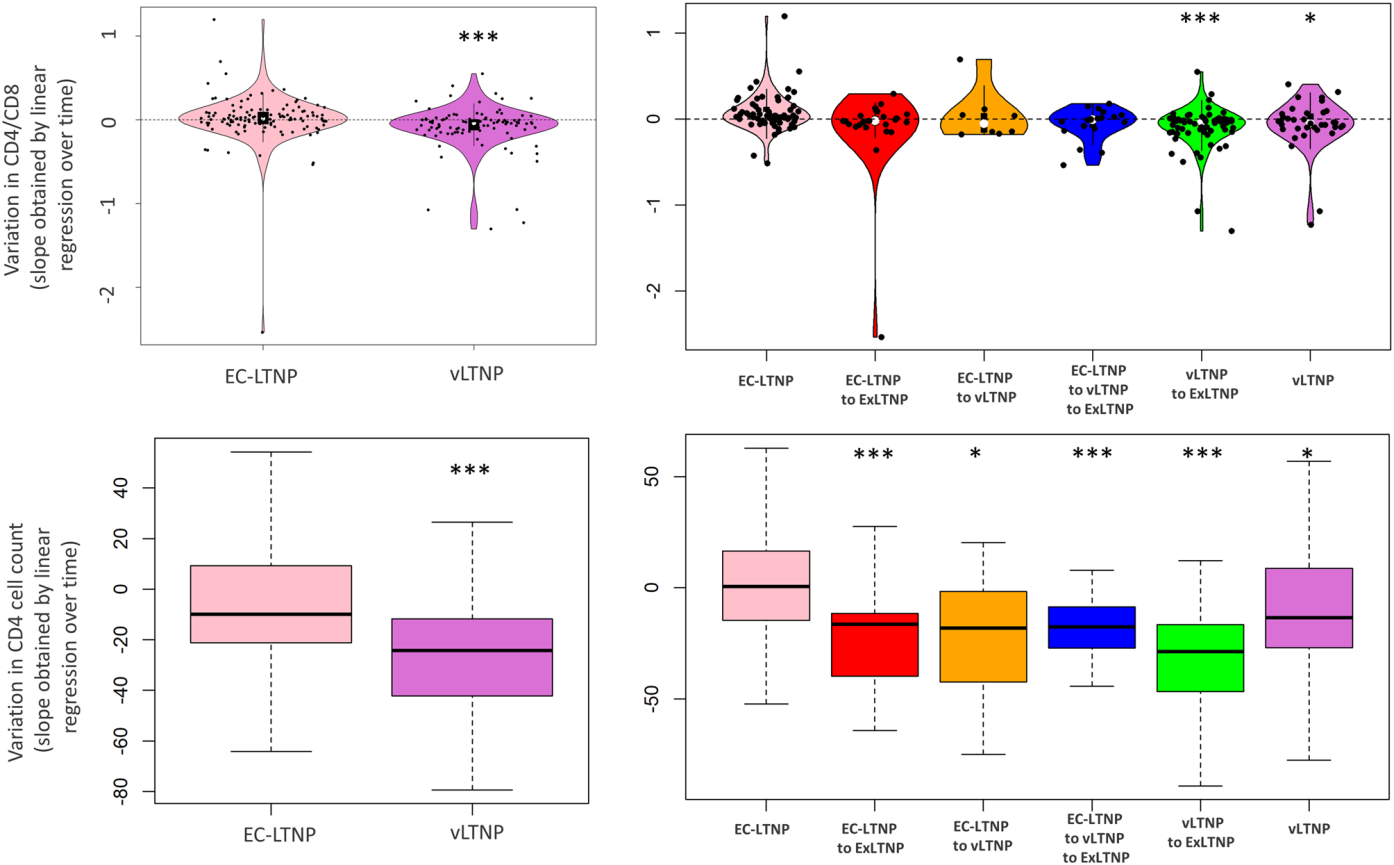
CD4 cell counts and CD4/CD8 during follow-up. Variation in CD4 cell counts and CD4/CD8 ratios was expressed as the slope obtained by linear regression over time in individuals primarily classified as EC-LTNP or vLTNP (left panels) or as EC-LTNP maintaining their status, EC-LTNP losing LTNP status, EC-LTNP re-classified as vLTNP during follow-up, EC-LTNP primarily re-classified as vLTNP and then losing LTNP phenotype, vLTNP maintaining their status and vLTNP losing LTNP phenotype (right panels). *p < 0.05; **p < 0.01; ***p < 0.001 compared to EC-LTNP or EC-LTNP maintaining the phenotype, respectively.

Statistically significant differences were found comparing globally the different phenotypes of LTNP status loss (*p* = 2.6 × 10^−13^). Specifically, pairwise comparisons determined statistically significant differences between EC-LTNP who maintain their phenotype and the two phenotypes of vLTNP, those who maintain the vLTNP status (− 13.5 cells/μl/year; IQR from − 26.4 to 8.7; *p* = 2.9 × 10^−2^) and those who loss LTNP phenotype (− 28.7 cells/μl/year; IQR from − 46.8 to − 16.7; *p* = 3.8 × 10^−14^). Also compared with EC-LTNP re-classified as vLTNP (− 18.2 cells/μl/year; IQR from − 42.4 to − 1.7; *p* = 4.95 × 10^−2^), EC-LTNP primarily re-classified as vLTNP and then losing LTNP status (− 17.7 cells/μl/year; IQR from − 27.0 to − 8.8; *p* = 4.5 × 10^−4^) and EC-LTNP losing LTNP phenotype directly (− 16.5 cells/μl/year; IQR from − 39.8 to − 11.6; *p* = 4.4 × 10^−4^) (Fig. 2).

The proportions of LTNP-RIS cohort participants with at least two measures of CD4/CD8 ratio was 120 out of the 163 EC-LTNP (73.6%) and 100 out of the 150 vLTNP (66.7%). Among EC-LTNP and vLTNP the median CD4/CD8 ratio was 0.93 (IQR = 0.80–1.08) and 0.65 (IQR 0.56–0.80), respectively (*p* < 2.2 × 10^−6^). The variation of CD4/CD8 estimated for these participants primarily classified as EC-LTNP and vLTNP was 0.01 per year (IQR from − 0.05 to 0.09) and − 0.09 per year (IQR from − 0.12 to 0.004), respectively (*p* = 2.0 × 10^−5^). Among EC-LTNP who maintain their status during the follow-up, the CD4/CD8 variation was 0.04 per year (IQR from − 0.01 to 0.13). Among EC-LTNP who lose their status during follow-up, the median CD4/CD8 ratio variation were: − 0.05 per year (IQR from − 0.15 to 0.06) for EC-LTNP re-classified as vLTNP, − 0.01 per year (IQR from − 0.10 to 0.04) for EC-LTNP primarily re-classified as vLTNP and then losing LTNP status, and − 0.02 per year (IQR from − 0.07 to 0.04) for EC-LTNP losing LTNP phenotype directly. Among vLTNP who maintain their status, the CD4/CD8 variation was − 0.05 per year (IQR from − 0.10 to 0.06) and − 0.03 per year (IQR from − 0.15 to 0.00) for vLTNP who lost LTNP status during follow-up (Fig. 2). Statistically significant differences were found comparing globally the different phenotypes of LTNP status loss (*p* = 1.4 × 10^−5^). Pairwise comparisons determined statistically significant differences between EC-LTNP who maintain the phenotype and the two phenotypes of vLTNP, those who maintain the vLTNP status (*p* = 2.1 × 10^−2^) and those who loss LTNP phenotype (*p* = 1.7 × 10^−6^).

### Variation of HIV VL during follow-up

**Figure 3:**
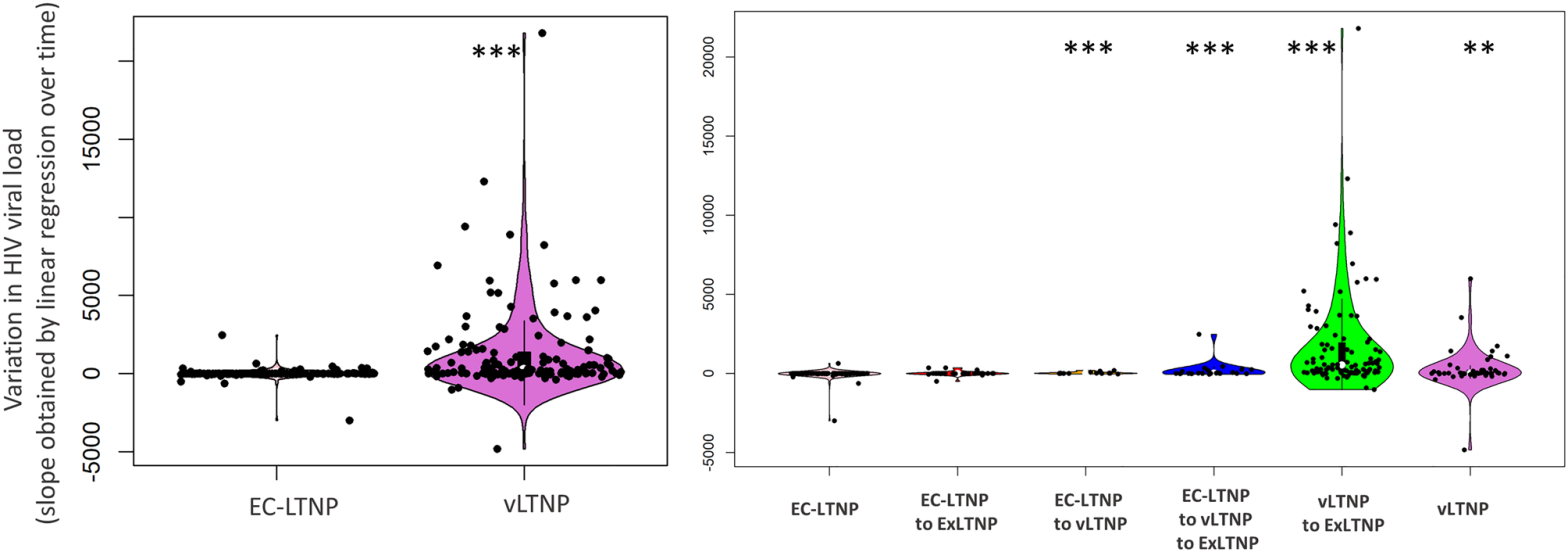
Variation of HIV VL during follow-up. Variation in HIV VL was expressed as the slope obtained by linear regression over time in individuals primarily classified as EC-LTNP or vLTNP (left panels) or as EC-LTNP maintaining their status, EC-LTNP losing LTNP status, EC-LTNP re-classified as vLTNP during follow-up, EC-LTNP primarily re-classified as vLTNP and then losing LTNP phenotype, vLTNP maintaining their status and vLTNP losing LTNP phenotype (right panels). Overall statistically significant differences were found in colored panels. *p < 0.05; **p < 0.01; ***p < 0.001 compared to EC-LTNP or EC-LTNP maintaining the phenotype, respectively.

The median VL for EC-LTNP during follow-up was 50 copies/mL (IQR = 40–50 copies/mL) compared with the 901.0 copies/mL (361.5–2301.2 copies/mL) observed in vLTNP (*p* = 2.9 × 10^−8^). The median variation of HIV VL in participants primarily classified as EC-LTNP and vLTNP was − 2.7 copies/mL/year (IQR from − 13.2 to 0.1) and 197.2 copies/mL/year (IQR from 12.3 to 1351.7), respectively (*p* < 2.2 × 10^−16^). Among EC-LTNP who maintain their status during the follow-up, the HIV VL variation was − 4.1 copies/mL/year (IQR from − 14.4 to − 0.9). Statistically significant differences were found comparing globally the different phenotypes of LTNP status loss (*p* < 2.2 × 10^−16^). Specifically, pairwise comparisons determined statistically significant differences between EC-LTNP who maintain their phenotype and the two phenotypes of vLTNP, those who maintain the vLTNP status (21.0 copies/mL/year; IQR from − 10.4 to 197.8; *p* = 1.4 × 10^−3^) and those who loss LTNP phenotype (534.8 copies/mL/year; IQR from 81.7 to 1933.2; *p* < 2.2 × 10^−16^), and also compared with EC-LTNP primarily re-classified as vLTNP and then losing LTNP status (17.3 copies/mL/year; IQR from − 2.4 to 178.9; *p* = 6.2 × 10^−4^) and EC-LTNP re-classified as vLTNP (11.2 copies/mL/year; IQR from 0.2 to 21.2; *p* = 7.8 × 10^−4^) (Fig. 3).

### Survival

**Figure 4:**
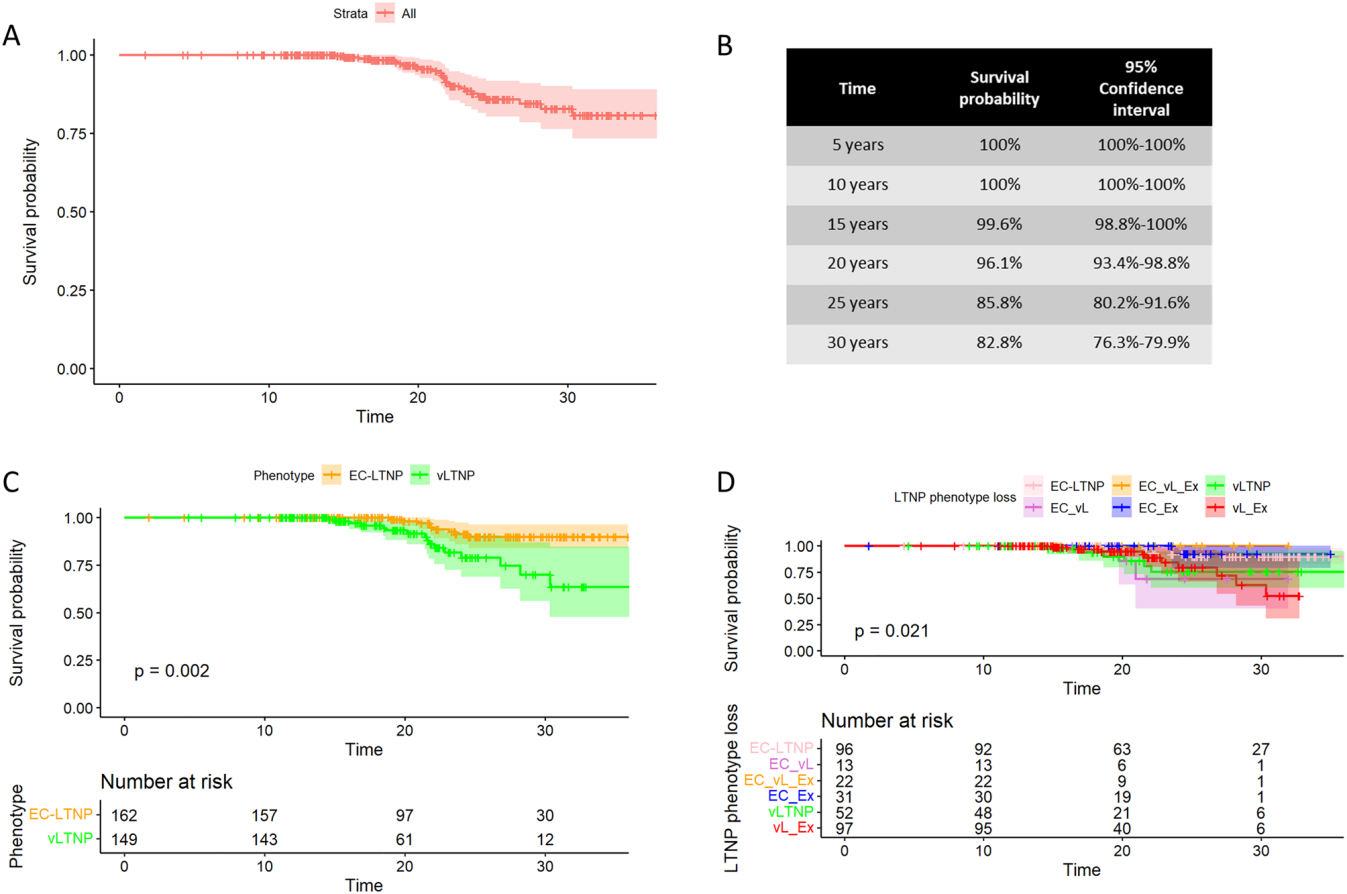
Probability to survive in LTNP-RIS cohort and hazard ratios. Kaplan–Meier estimates for the entire cohort (**A**) and associated survival probabilities (**B**). Kaplan–Meier estimates comparing EC-LTNP and vLTNP populations (**C**). Kaplan–Meier estimates comparing the different types of LTNP phenotype loss (**D**). Time is expressed in Kaplan-Meir curves as years from HIV + diagnosis.

During follow-up, 25 individuals died, including 9 EC-LTNP and 16 vLTNP. AIDS-defining conditions were reported in two EC-LTNP, including a case of pulmonary tuberculosis and a progressive multifocal leukoencephalopathy; and in three vLTNP, including two cases of pulmonary tuberculosis and one case of Kaposi´s sarcoma. The causes of death were reported in 16 out of the 25 cases and were mainly associated to different types of cancer, but also to neurological, infectious and inflammatory diseases and also to one accidental death and a suicide. The estimated survival probability at a given time since diagnosis for the LTNP-RIS cohort was 100% at 10 years, 96.1% (95% CI 93.4–98.8%) at 20 years and 82.8% (95% CI 76.3–89.9%) at 30 years (Fig. 4). AIDS-defining conditions, such as pulmonary tuberculosis, progressive multifocal leukoencephalopathy, and Kaposi´s sarcoma among others, were described for 10 EC-LTNP and 16 vLTNP. No statistically significant differences in CD4 counts, viral loads, or CD4:CD8 ratios were found between all EC-LTNP individuals and those who died/developed AIDS or between all vLTNP individuals and those who died/developed AIDS.

Statistically significant differences were found comparing the survival curves for EC-LTNP and vLTNP (*p* = 2.0 × 10^−3^). The estimated survival probabilities were 100% for both phenotypes at 10 years, 98.1% (95% CI 95.5–100%) for EC-LTNP and 93.3% (95% CI 88.2–98.7%) for vLTNP at 20 years and 90.0% (95% CI 84.0–96.5%) for EC-LTNP and 70.0% (95% CI 56.3–87.1%) for vLTNP at 30 years. Loss of LTNP phenotype was also associated to survival (*p* = 0.02), with estimated survival probabilities at 30 years of 90.0% (82.7–98.0%) for EC-LTNP maintaining the phenotype, 68.6% (40.3–100.0%) for EC-LTNP reclassified as vLTNP, 100% for EC-LTNP initially reclassified as vLTNP and later as ExLTNP, 92.3% (78.9–100.0%) for EC-LTNP losing LTNP phenotype directly, 75.6% (59.9–95.4%) for vLTNP maintaining the phenotype, and 62.7% (42.7–92.0%) for vLTNP losing LTNP phenotype. No differences were found in survival comparing HCV seronegative individuals with HCV coinfected individuals. The hazard of death in univariable analyses was associated with the initial vLTNP condition (*p* = 3.6 × 10^−3^), including vLTNP maintaining the phenotype (*p* = 0.03) and vLTNP losing LTNP phenotype during follow up (*p* = 1.6 × 10^−2^) (Fig. 4). The multivariate analysis pointed out the association of the initial EC-LTNP phenotype with a decrease in the hazard of death in the LTNP-RIS cohort participants (HR = 0.28; 95% CI 0.12–0.65; *p* = 0.02).

### Disease progression

Participants primarily classified as EC-LTNP suppose 52.1% of LTNP-RIS, including a 60.0% of all women and a 47.8% of all men, and those individuals primarily classified as vLTNP constitute 47.9% of LTNP-RIS, including 40.0% of all women and 52.2% of all men. Some of these participants primarily classified as LTNP lost their status due to different causes, including the initiation of ART, a decrease in CD4 T cell count to below 500 cells per μl (immunologic progression), and/or an increase in HIV VL above 10,000 copies/mL (virologic progression). LTNP-RIS cohort includes 53 EC-LTNP (32.5% of all EC-LTNP) and 98 vLTNP (65.3% of all vLTNP) which lose LTNP phenotype (*p* = 1.3 × 10^−7^). In the case of ExLTNP initially classified as EC-LTNP, a median CD4 count of 468 cells/μl (IQR = 367–608 cells/μl) and a median viral load of 246 copies/mL (IQR = 64–2134 copies/mL) were detected just before the initiation of ART. On his part, vLTNP becoming ExLTNP showed a median CD4 count of 410 cells/μl (IQR = 285–578 cells/μl) and a median viral load of 8518 copies/mL (IQR = 1075–25,553 copies/mL) before the initiation of ART. Only 13 EC-LTNP were reclassified as vLTNP during follow-up (8.0% of all EC-LTNP) because of an increase in HIV VL.

A total of 29 AIDS-defining opportunistic infections were detected in 24 cohort participants, including pulmonary tuberculosis (n = 11), progressive multifocal leukoencephalopathy caused by JC papovavirus infection (n = 4), esophageal candidiasis (n = 3), bacterial recurrent pneumonia (n = 3), *Pneumocystis jirovecii* pneumonia (n = 2), Kaposi´s sarcoma caused by HHV-8 infection (n = 2), HIV encephalopathy (n = 2), and HPV cervical cancer (n = 2). Although the frequency in EC-LTNP who maintain the phenotype during follow-up was the lower found in the cohort, the differences with vLTNP (4.1% versus 9.6%, *p* = 0.28) and with those losing LTNP condition were not statistically significant (4.1% versus 9.4%, *p* = 0.28). There was also no difference comparing the vLTNP who maintain the phenotype with those that lose it (9.6% versus 10.2%, *p* = 1.0).

**Figure 5:**
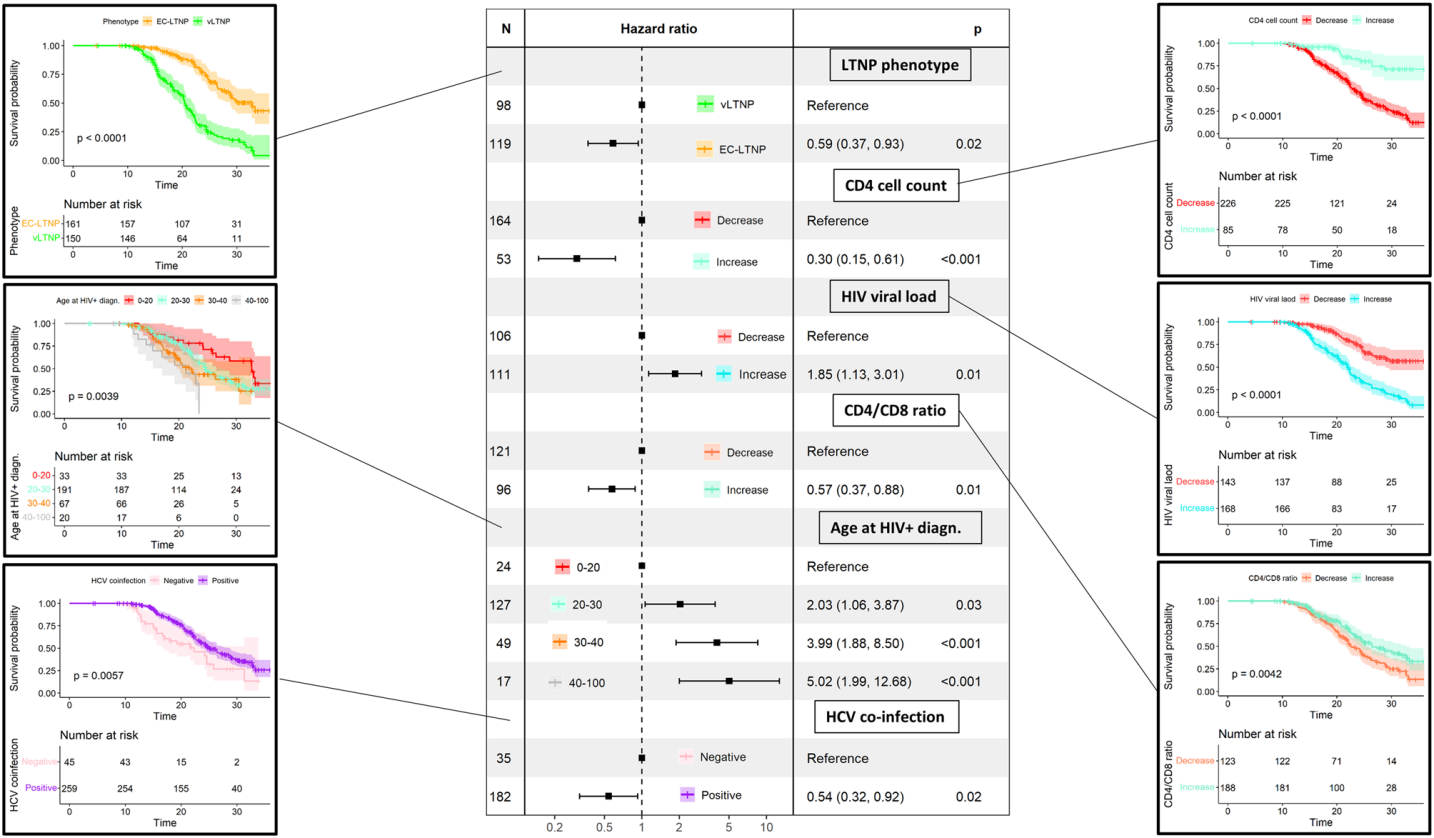
Probability of retaining LTNP phenotype in LTNP-RIS cohort participants and hazard ratios of significant variables. Kaplan–Meier estimates of the probability of retaining LTNP phenotype of the statistically significant covariates in univariate analyses (LTNP phenotype, age at HIV + diagnosis, HCV coinfection and variations in CD4 cell count, HIV VL and CD4/CD8 ratio). Determinants associated with loss of LTNP phenotype in a multivariate Cox proportional hazards model are showed in the middle. Time is expressed in years from HIV + diagnosis.

Differences in the progression-free survival curves of EC-LTNP and vLTNP were identified (*p* < 2.2 × 10^−16^). The estimated progression-free probabilities were 100% for EC-LTNP and vLTNP at 10 years, 88.4% (95% CI 83.1–93.9%) for EC-LTNP and 57.2% (95% CI 49.2–66.6%) for vLTNP at 20 years and 50.5% (95% CI 40.9–62.3%) for EC-LTNP and 17.9% (95% CI 11.5–27.8%) for vLTNP at 30 years. Regarding the different phenotypes of loss of LTNP status, the estimated progression-free probabilities at 20 years were 80.9% (95% CI 65.8–99.6%) for EC-LTNP primarily re-classified as vLTNP and then losing LTNP status, 61.3% (95% CI 46.3–81.1%) for EC-LTNP losing LTNP status directly, and 43.9% (95% CI 35.1–54.9%) for vLTNP who lost LTNP status. These differences in the estimated progression-free probabilities between groups of individuals augmented at 30 years, reaching 9.5% (95% CI 2.6–35.6%), 3.2% (95% CI 0.5–22.2%), and 5.1% (95% CI 2.2–12.0%) for EC-LTNP primarily reclassified as vLTNP and then losing LTNP status, EC-LTNP losing LTNP phenotype directly, and vLTNP losing LTNP phenotype, respectively (Fig. 5).

Differences in progression-free curves was also observed comparing HCV − and HCV + (*p* = 6.0 × 10^−3^), reaching an estimated progression-free probabilities at 10 years of 100% for both groups, of 54.7% (40.8–73.5%) for HCV- and 76.5% (71.2–82.3%) for HCV + at 20 years and 26.8% (13.8–52.0%) for HCV- and 36.5% (29.7–44.9%) for HCV + at 30 years. Age at HIV + diagnosis was also considered a significant variable (*p* = 4.0 × 10^−3^), with estimated progression-free probabilities at 20 years of 81.6% (69.3–96.1%) for the younger group of participants diagnosed before 20 years old, 78.1% (72.0–84.8%) for participants diagnosed between 20 and 30 years old, 61.2% (49.6–75.5%) for those diagnosed between 30 and 40 years old, and 53.6% (32.9–87.4%) for those diagnosed after 40 years old. Estimated variations in CD4 cell counts (*p* = 7.0 × 10^−9^), CD4/CD8 cell ratio (*p* = 4.0 × 10^−3^), and VL (*p* = 1.0 × 10^−11^) were also considered statistically significant variables in progression-free curves. On the other hand, no statistically significant differences were found for sex and transmission route.

The factors associated to the loss of LTNP phenotypes in univariate analyses were the initial vLTNP phenotype (*p* = 2.5 × 10^−14^), the CD4 T cell count loss (*p* = 1.3 × 10^−7^), the increase of HIV VL (*p* = 1.5 × 10^−10^), the decrease in the CD4/CD8 ratio (*p* = 5.2 × 10^−5^), and the age at HIV + diagnosis, including the individuals becoming HIV + in their thirties (*p* = 1.1 × 10^−2^) and in people older than 40 (9.8 × 10^−4^), and the absence of HCV coinfection (*p* = 6.5 × 10^−3^). Protective factors associated with non-progression phenotypes in the multivariate analyses were the initial EC-LTNP phenotype (HR = 0.59, 95% CI = 0.37–0.93; *p* = 2.4 × 10^−2^), the increase in both CD4 cell counts (HR = 0.30, 95% CI = 0.15–0.61; *p* = 9.1 × 10^−4^) and CD4/CD8 ratio (HR = 0.57, 95% CI = 0.37–0.88; *p* = 1.1 × 10^−2^) during follow-up, and HCV coinfection (HR = 0.54, 95% CI = 0.32–0.92; *p* = 2.4 × 10^−2^). The risk factors found in the model were an increase in HIV VL during follow-up (HR = 1.85, 95% CI = 1.13–3.01; *p* = 1.4 × 10^−2^) and becoming HIV + in the twenties (HR = 2.03, 95% CI = 1.06–3.87; *p* = 3.2 × 10^−2^), or in the thirties (HR = 3.99, 95% CI = 1.88–8.50; *p* = 3.2 × 10^−4^), or older than 40 (HR = 5.02, 95% CI = 1.99–12.68; *p* = 6.3 × 10^−4^).

## Discussion

Most participants in LTNP-RIS cohort are from Spain (95.5%) and the main route of transmission was the injection drug use (IDU) which counts with 75.4% of cases. This reflects the epidemic characteristics in Spain at the time most of them became infected. In the nineties, 70% of AIDS patients in Spain were IDU^4^, and this high proportion of IDU among patients was a distinctive feature of the epidemic in Spain. In addition, as blood is the most effective route of transmission of HCV, a high percentage of the participants in the cohort (82.7%) were coinfected with HCV including a 96.2% of all IDU, as observed in previous studies^5,6^. Two thirds of participants (64.9%) are men, reflecting the higher proportion of infected men compared to women in the epidemic in Spain along time.

The main defining criteria of the LTNP cohort is the immunological one, in this sense, cohort participants are defined as those maintaining levels above 500 CD4 cells/µl for more than 10 years without antiretroviral treatment. Regarding virological criteria, LTNP must have a VL below 10,000 copies/mL, which includes two different participant profiles: EC- LTNP and vLTNP. Approximately half of our participants were EC-LTNP (52.1%) and the other half were vLTNP (47.9%), and, as previously described, among women, the probability of being EC-TNP is higher than among men^7–9^. The percentage of HCV coinfection in EC-LTNP and vLTNP is similar, 85.9% and 79.3% respectively, which corresponds to similar proportions of participants infected by injection drug use in both groups (77.2% in EC-LTNP and 74.0% in vLTNP).

The initial classification as EC-LTNP was the only variable associated with a decrease in the hazard of death in the LTNP-RIS cohort participants. Some studies defend that elite controllers (EC) seems to be at a higher risk of all causes of hospitalisation compared with HIV + individuals on ART, as a consequence of a low-grade persistent inflammation^9,10^. These studies have evaluated the main hospitalisation causes among EC, indicating that cardiovascular and psychiatric diseases are the most frequent in this population compared with different phenotypes of HIV + treated non-controllers. Specifically, coronary atherosclerosis and heart failure are among the most common diagnoses, along with higher levels of soluble(s)-CD163 in EC compared with HIV- and HIV + treated non-controllers^10^. The plasma level of sCD163 is considered a monocyte activation marker and a predictor of all-cause mortality in HIV + individuals^11^. However, the lack of a control group of viremic controllers/vLTNP in these studies makes these results not comparably to our study. In this sense, Okulicz et al. (2009) studied the disease progression in a military cohort of 4586 PLWH and found that EC had longer time to AIDS diagnosis and substantially more favourable time to development of a CD4 cell count threshold of 350 cells/μL than viremic controllers^12^. Although the definition of EC in this study is not comparably to our definition of EC-LTNP due to our stricter parameters in terms of longer periods of HIV control, it seems that both studies confirm that viral suppression of virus replication observed in EC is beneficial in terms of survival and disease progression.

As expected, there are differences in the median variation of HIV viral load during follow-up comparing patients primarily classified as EC-LTNP and vLTNP, with a slight decrease in EC-LTNP and an increase in vLTNP, and there are statistically significant differences comparing globally the different phenotypes of LTNP status loss. As CD4 + lymphocytes are the main target for HIV, expectable differences are observed in the variations of CD4+ T cells during the follow-up when comparing EC- LTNP and vLTNP. The median variation of CD4 counts in participants primarily classified as EC-LTNP was − 9.9 cells/μl/year and − 24.2 in vLTNP. EC-LTNP who maintain their status showed no loss of CD4 T cells during the follow-up.

CD4/CD8 ratio inversion has been described as a consequence of HIV infection due to depletion of CD4 T cells and expansion of HIV-specific and nonspecific CD8 T cells, and this rate inversion is not always reverted by effective ART, in spite of CD4 recovery^13^. HIV-infected individuals with an inverted CD4/CD8 ratio (< 1.0) are characterized by immune activation and immunosenescent phenotype, and higher risk of morbidity/mortality, and this clinical scenario is also observed in HIV uninfected elderly adults, and is characterized by a low CD4/CD8 ratio^14,15^. In our cohort, the median CD4/CD8 ratio was close to 1.0 (0.93; IQR = 0.67–1.28) in EC-LTNP and 0.65 (IQR = 0.45–0.89) for vLTNP. The global variation of CD4/CD8 estimated for EC-LTNP was 0.01 and − 0.09 for vLTNP per year, which correspond, at least in part, with different variations of CD4 during the follow-up observed in both groups. Therefore, in addition to CD4, the classical clinical marker of HIV disease progression, CD4/CD8 ratio is another suitable marker of progression in these patients, and can detect individuals with immunological abnormalities and worse prognosis in spite of high levels of CD4^16^.

The median duration of the LTNP status in the 52 LTNP identified in a military cohort of 4586 PLWH was around 12 years^12^, compared with the median of 23.6 years (IQR = 18.7–28.8) of the 162 EC-LTNP and the 18.2 years (IQR = 14.9–22.2) of the 151 vLTNP of the present study. Other cohort studies of HIV seroconverters showed lower median times to loss LTNP status, with values between 12 years obtained in both the CASCADE Collaboration network (with cohorts from Europe, Australia, Canada, and sub-Saharan Africa) and the French ANRS SEROCO/HEMOCO cohort, and the 14 years estimated in the San Francisco City Clinic Study^17–19^. Another work associated viral blips to higher risk of immunological progression in EC, which is consistent with the results observed in the present work comparing EC-LTNP and vLTNP capabilities to maintain LTNP phenotype^20^. Specifically, in this study and in the present work, the lasting loss of CD4+ T cells and the residual HIV replication were statistically significant variables associated to disease progression^20^. Thus, the combination of EC and LTNP phenotypes observed in EC-LTNP appears to be the most optimal form of immune activation found in nature to cope HIV-1 infection in terms of prevention of AIDS and death.

Age at diagnosis was also associated to the loss of non-progression in the present study. Specifically, HIV + diagnosis in their twenties, thirties and more than 40 years old was associated to a 2.0-, 4.0- and 5.0-increase in the hazard of LTNP status loss, respectively, and compared to individuals diagnosed before 20 years old. This association of age with the loss of LTNP status was also described by van der Helm et al. (2014) in a cohort of 4979 HIV-seroconverters^20^. In this study, however, the authors suggested that after a period of 10 years free of progression, age is no longer significantly associated with progression. Age itself induces changes in the number, proportion and function of CD4+ T cells, including a reduction in the naïve lymphocyte population, a decrease in T-cell receptor (TCR) diversity, and the incapacity to replace CD4+ T cell population by thymic involution^13–15^. This situation leads to an exhaustion of some T-lymphocytes clone and the incapacity to maintain the memory pool with aging, resulting in a decreased ability to respond to pathogens. Moreover, as discussed before, HIV-1 infection triggers a premature aging response, including neurologic impairment, metabolic disorders, immunosenescence and bone diseases^21,22^. All these studies argue that age is strongly associated to HIV-1 disease progression and therefore, the loss of LTNP phenotype occurs more rapidly in older than in younger PLWH.

HCV coinfection was associated to the conservation of LTNP phenotype in our study. A plausible factor explaining this result could be the beneficial effect of HCV-specific therapies based on interferon, including pegylated interferon/ribavirin with or without boceprevir, telaprevir or direct-acting antivirals. Within LTNP-RIS cohort, more than half of HCV-coinfected individuals have received this type of treatments anytime (51.6%). These HCV-specific therapies based on interferon were the unique therapy option for the cohort participants until the end of 2013 and the beginning of 2014, date when interferon-free direct-acting antivirals such as sofosbuvir and simeprevir were authorized for use in the European Union by the European Medicine Agency (EMA) and in the USA by the U.S. Food and Drug Administration (FDA). Regarding HIV-specific treatment, the majority of LTNP-RIS participants were diagnosed as HIV + in the pre-ART era (86.9% before the approval in 1996 of the first protease inhibitor, named saquinavir). On the other hand, HCV infection itself leads to the induction of type I interferon through the recognition of viral RNA by the endosomal sensors TLR3, 7 and 8^23^. In the context of the LTNP-RIS cohort composed by a high proportion of HCV co-infected individuals receiving no therapy for HIV, interferon-based treatments and the innate immune response against HCV infection could have a positive effect suppressing retrovirus replication in LTNP by stimulating the expression of HIV restriction factors^24^. However, the beneficial effect of interferon in HIV/HCV co-infected individuals is certainly controversial^24^. Another study focused on LTNP population co-infected with HCV genotype 3a found an increased risk of losing LTNP status compared with patients infected with HCV genotype 1 (HR = 2.3; 95% CI = 1.1–4.8)^25^. Although this trend of a higher presence of HCV genotype 1 among EC-LTNP who maintain their phenotype was also observed in the LTNP-RIS cohort, no statistically significant differences were observed in this sense.

In conclusion, the LTNP-RIS cohort includes participants mainly infected by injection drug use and, for this reason, a high percentage of them are coinfected with HCV. Clinical markers reflect a better prognosis in EC-LTNP compared to vLTNP: lower decrease of CD4 counts, better CD4/CD8 ratio and even a slight decrease in median variation of HIV VL during follow-up, longer median duration of the LTNP status and survival. Thus, EC-LTNP individuals, characterized by combining a preserved level of CD4 and suppressed VL, seems to be an excellent model of functional cure found in nature. Even more so after the appearance of some cases of long-term virological control observed in EC-LTNP and considered as possible cases of HIV sterilizing cure^26–28^.

## Data Availability

Personal medical histories analysed during the current study are not publicly available due to the policy on privacy and data protection but are available from the corresponding authors on reasonable requests and after the approval of the LTNP-RIS cohort committee.
